# Involvement of the Precuneus/Posterior Cingulate Cortex Is Significant for the Development of Alzheimer’s Disease: A PET (THK5351, PiB) and Resting fMRI Study

**DOI:** 10.3389/fnagi.2018.00304

**Published:** 2018-10-05

**Authors:** Takamasa Yokoi, Hirohisa Watanabe, Hiroshi Yamaguchi, Epifanio Bagarinao, Michihito Masuda, Kazunori Imai, Aya Ogura, Reiko Ohdake, Kazuya Kawabata, Kazuhiro Hara, Yuichi Riku, Shinsuke Ishigaki, Masahisa Katsuno, Shinichi Miyao, Katsuhiko Kato, Shinji Naganawa, Ryuichi Harada, Nobuyuki Okamura, Kazuhiko Yanai, Mari Yoshida, Gen Sobue

**Affiliations:** ^1^Department of Neurology, Nagoya University Graduate School of Medicine, Nagoya, Japan; ^2^Brain and Mind Research Center, Nagoya University, Nagoya, Japan; ^3^Department of Neurology, Meitetsu Hospital, Nagoya, Japan; ^4^Department of Radiological and Medical Laboratory Sciences, Nagoya University Graduate School of Medicine, Nagoya, Japan; ^5^Department of Radiology, Nagoya University Graduate School of Medicine, Nagoya, Japan; ^6^Department of Pharmacology, Tohoku University School of Medicine, Sendai, Japan; ^7^Division of Pharmacology, Faculty of Medicine, Tohoku Medical and Pharmaceutical University, Sendai, Japan; ^8^Department of Neuropathology, Institute for Medical Science of Aging, Aichi Medical University, Nagakute, Japan

**Keywords:** ^18^F-THK5351, ^*11*^C-PiB, positron emission tomography (PET), resting state network, MRI, MAO-B, astrocyte

## Abstract

**Background**: Imaging studies in Alzheimer’s disease (AD) have yet to answer the underlying questions concerning the relationship among tau retention, neuroinflammation, network disruption and cognitive decline. We compared the spatial retention patterns of ^18^F-THK5351 and resting state network (RSN) disruption in patients with early AD and healthy controls.

**Methods**: We enrolled 23 ^11^C-Pittsburgh compound B (PiB)-positive patients with early AD and 24 ^11^C-PiB-negative participants as healthy controls. All participants underwent resting state functional MRI and ^18^F-THK5351 PET scans. We used scaled subprofile modeling/principal component analysis (SSM/PCA) to reduce the complexity of multivariate data and to identify patterns that exhibited the largest statistical effects (variances) in THK5351 concentration in AD and healthy controls.

**Findings**: SSM/PCA identified a significant spatial THK5351 pattern composed by mainly three clusters including precuneus/posterior cingulate cortex (PCC), right and left dorsolateral prefrontal cortex (DLPFC) which accounted for 23.6% of the total subject voxel variance of the data and had 82.6% sensitivity and 79.1% specificity in discriminating AD from healthy controls. There was a significant relationship between the intensity of the ^18^F-THK5351 covariation pattern and cognitive scores in AD. The spatial patterns of ^18^F-THK5351 uptake showed significant similarity with intrinsic functional connectivity, especially in the PCC network. Seed-based connectivity analysis from the PCC showed significant decrease in connectivity over widespread brain regions in AD patients. An evaluation of an autopsied AD patient with Braak V showed that ^18^F-THK5351 retention corresponded to tau deposition, monoamine oxidase-B (MAO-B) and astrogliosis in the precuneus/PCC.

**Interpretation**: We identified an AD-specific spatial pattern of ^18^F-THK5351 retention in the precuneus/PCC, an important connectivity hub region in the brain. Disruption of the functional connections of this important network hub may play an important role in developing dementia in AD.

## Introduction

The pathological hallmarks of Alzheimer’s disease (AD) are the presence of extracellular senile plaques containing amyloid β (Aβ) peptide, and intracellular neurofibrillary tangles of the microtubule-associated protein tau (Montine et al., 2012; Canter et al., 2016). Astrogliosis associated with neurodegeneration is also a major hallmark of AD (Rodríguez-Arellano et al., 2016). However, the roles of Aβ, tau and astrogliosis/neurodegeneration in developing dementia have not been fully elucidated.

According to the Nun study, some advanced Braak’s stage subjects with a significant increase in tau and Aβ accumulation showed good cognition during their lives (Snowdon, 1997), suggesting that brain neuronal networks may play an important role in the maintenance of cognition or development of dementia. Consequently, combined use of Aβ/tau PET and functional MRI to investigate brain networks in humans is expected to identify a convincing relationship between these pathological changes and the cognitive declines in AD.

Previous studies combining amyloid PET and resting-state functional MRI (rsfMRI) have demonstrated several important findings regarding the relationship between Aβ retention and resting state network (RSN) changes in AD: (1) Aβ retention in the neocortex regions is associated with cognition and spreads to hub regions corresponding to nodes of the default mode network (DMN; Lustig et al., 2003; Greicius et al., 2004); (2) increased Aβ retention alters task-related fMRI signal response in the DMN (Sperling et al., 2009; Mormino et al., 2011; Lim et al., 2014); and (3) cortical Aβ retention is also associated with disrupted RSN of the perirhinal and precuneus cortex (Song et al., 2015) and decreased connectivity to various anatomical lesions (Sheline et al., 2010). However, in these studies, tau, astrogliosis and neuroinflammation were not taken into account.

Despite cognitive deterioration and progressive loss of intra/internetwork connectivity at the DMN and other RSNs in association with the progression of AD (Agosta et al., 2012; Binnewijzend et al., 2012; Brier et al., 2012; Damoiseaux et al., 2012), the Aβ retention levels remain almost stationary over the conversion period from the mild cognitive impairment (MCI) state to AD (Jack et al., 2009). In addition, Aβ retention cannot fully explain the clinical-anatomical heterogeneity in AD (Ranasinghe et al., 2014; Lehmann et al., 2015; Canter et al., 2016). Finally, most Aβ-based antibody treatments that eradicate Aβ retention failed to restore cognitive function (Sacks et al., 2017; Wang et al., 2017).

These observations raise the question regarding the critical role of Aβ deposition, tau deposition and astrogliosis/neurodegeneration, particularly in terms of their spatial distribution, temporal timing and relationship to brain network disruption, in the development of the dementia in AD.

The spatial spread and temporal pattern of THK5351 retention correspond to the known distribution of tau pathology associated with the clinical severity and symptomatology of cognitive decline (Harada et al., 2016). However, recent studies have demonstrated that THK5351 also binds to monoamine oxidase-B (MAO-B), which is mainly localized in the inner mitochondrial membrane of astrocytes and increases with astrogliosis (Levitt et al., 1982; Ekblom et al., 1993; Lemoine et al., 2017; Ng et al., 2017; Harada et al., 2018). Regional *in vivo* THK5351 retention was significantly correlated with the density of tau aggregates in the neocortex and MAO-B in the whole brain (Harada et al., 2018). Furthermore, a significant association was observed between the density of tau aggregates, MAO-B and glial fibrillary acidic protein (GFAP), suggesting that neocortical tau strongly influences the formation of reactive astrocytes (Harada et al., 2018). In AD, astrogliosis and microglial activations progress together with tau pathology and contribute to neurodegeneration throughout the course of the disease (Leyns and Holtzman, 2017). Thus, THK5351 retention in the AD neocortex is expected to evaluate the spatial distribution of tau pathology and astrogliosis/neurodegeneration in the human brain.

In this study, we analyzed the AD-related spatial THK5351 distribution pattern using scaled subprofile modeling/principal component analysis (SSM/PCA), an unbiased data-driven approach, and investigated the relationship between THK5351 retention and RSN involvement. In addition, we investigated the pathological findings, including tau, MAO-B and activated astrocytes, of AD-related regions. Based on these approaches, we demonstrated the significance of the obtained spatial pattern of THK5351 and its relevance to network involvement, which are critical to the development of AD.

## Participants and Methods

### Participants

We enrolled 63 participants (36 early AD patients, 27 healthy controls) in this study. All participants underwent MRI, Aβ PET imaging with ^11^C-Pittsburgh compound B (PiB), PET imaging with THK5351 and cognitive functional testing, which included the mini mental state examination (MMSE), the Addenbrooke’s Cognitive Examination Revised (ACE-R; Mioshi et al., 2006), AD Assessment Scale-Cognitive-Japanese (ADAS-cog-j), and Logical Memory II of the Wechsler Memory Scale Revised, Clinical Dementia Rating (CDR) and CDR Scale Sum of Boxes (CDR-SB).

All patients with early AD were recruited from the outpatient clinic of the Department of Neurology, Nagoya University Hospital, and Dementia Center of Meitetsu Hospital in Nagoya. There were no patients with atypical features suggestive of variant types of AD (posterior cortical atrophy, logopenic aphasia, or frontal-variant), significant medical illness, history of brain trauma, brain surgery, or evidence of other neurological disease. The criteria for diagnosis of early AD were as follows: (1) memory complaint; (2) 0.5 or 1.0 in CDR; (3) a score lower than one standard deviation (SD) minus the average of their ages in Logical Memory II; and (4) PiB positive as described later. All patients fulfilled the National Institute of Neurologic and Communicative Disorders and Stroke and the AD Related Disorders Association (NINCDS-ADRDA; McKhann et al., 2011) or Peterson’s MCI criteria (Petersen, 2004). Clinical diagnoses were made with the consensus of three neurologists (HW, SM and GS).

Cognitively normal controls were recruited from a healthy cohort aging study by the Brain and Mind Research Center of Nagoya University. The participants in the control group had no history of neurologic or psychiatric illness, no abnormalities on neurologic examination, no subjective memory complaints, a CDR score of 0, an MMSE score of 26 or higher, an ACE-R score of 89 or higher, and a score higher than minus one SD of the average of their ages in the Logical Memory II of the Wechsler Memory Scale Revised. No focal deep white matter (WM) abnormalities characterized by hyperintensities more severe than grade 2 of the Fazekas hyperintensity rating system were observed in T2-weighted MR images (Fazekas et al., 1991).

Based on these criteria, we excluded 13 of 36 early AD patients (nine were PiB negative, three had WM abnormalities or stroke regions, one had artifacts on MRI caused by dental restorations) and 3 of 27 healthy control (two were PiB positive, one had a low Logical Memory II score). Finally, 23 early AD patients and 24 healthy controls were included.

This study conformed to the Ethical Guidelines for Medical and Health Research Involving Human Subjects endorsed by the Japanese government and received approval from the Research Ethics Committee of Nagoya University School of Medicine. All participants provided both informed and written consent to participate in this study and were treated according to the Declaration of Helsinki.

### MRI Study

All MRI scans were performed using a Siemens Magnetom Verio (Siemens, Erlangen, Germany) 3.0 T scanner with a 32-channel head coil at Nagoya University’s Brain and Mind Research Center. High-resolution T1-weighted images (Repetition Time (TR) = 2.5 s, Echo Time (TE) = 2.48 ms, 192 sagittal slices with 1-mm thickness, field of view (FOV) = 256 mm, 256 × 256 matrix size) were acquired for anatomical reference. rsfMRI scans (8 min, eyes closed) were also acquired (TR = 2.5 s, TE = 30 ms, 39 transverse slices with a 0.5-mm inter-slice interval and 3-mm thickness, FOV = 192 mm, 64 × 64 matrix dimension, flip angle = 80 degrees).

### Voxel-Based Morphometry (VBM)

The T1-weighted images were preprocessed using statistical parametric mapping (SPM) 12 (Wellcome Trust Center for Neuroimaging, London, UK^1^) running on Matlab (R2016b, Math Work, Natick, MA, USA). The images were first segmented into component images that included gray matter (GM), WM and cerebrospinal fluid (CSF). The segmented images were then processed using DARTEL (Ashburner, 2007) to obtain a group template, normalized to the standard Montreal Neurological Institute (MNI) space, resampled to an isotropic 2 × 2 × 2 mm^3^ voxel resolution, and smoothed using an 8-mm full-width-at-half-maximum (FWHM) isotropic 3D Gaussian filter. A two-sample *t*-test was performed to compare the processed images from the patient group to that of the healthy control group. We included age, gender and the total intracranial volume computed as the sum of GM, WM and CSF volumes, as covariates of no interest. The resulting statistical maps were thresholded using *p* < 0.05 corrected for multiple comparison using family-wise error (FWE) correction.

### Resting State fMRI Data Preprocessing

The resting state fMRI data were also preprocessed using SPM 12. For each participant’s data, the first five volumes were removed to account for the initial image inhomogeneity. The remaining images were then slice-time corrected, realigned relative to the mean functional image, co-registered to the participant’s anatomical image, normalized to the standard MNI space, resampled to a 2 × 2 × 2 mm^3^ voxel size, and finally, smoothed using an 8-mm FWHM 3D Gaussian filter. The six estimated motion parameters, mean signals from selected regions-of-interest (ROIs) within WM and CSF, and the corresponding forward (*t* + 1) and backward (*t* − 1) translations of these signals were also regressed to minimize the effects of head motion and other physiological noise. Finally, the processed images were bandpass filtered within the frequency range from 0.01 Hz to 0.1 Hz.

### Canonical RSN Analysis

Changes in well-known RSN were investigated using group independent component analysis (ICA) and dual regression analysis (Filippini et al., 2009). We used the MELODIC software from FSL package^2^ for ICA (Jenkinson et al., 2002, 2012; Smith, 2002). Almost 30 independent components were extracted and independent components with greatest overlap to RSN templates (Shirer et al., 2012) were identified. Subject-specific RSNs were computed using dual regression analysis (Filippini et al., 2009). Statistical analysis of the different RSNs was performed using nonparametric permutation testing (Nichols and Holmes, 2002) with 5,000 permutations to identify regions showing statistically significant differences in connectivity between the patient group and the healthy control group. A threshold-free cluster enhancement (TFCE) technique (Smith and Nichols, 2009) was used, and all statistical maps were corrected for multiple comparisons using FWE correction with *p* < 0.05.

### Seed Based Connectivity Analysis

To perform seed-based connectivity analysis, we generated several seed ROIs which showed the most significant differences in THK5351 retention between AD and healthy controls (see list below). Time series obtained by fMRI within each ROI were extracted, and the mean was computed. To obtain the connectivity between a given ROI and the whole brain, the correlation between the resulting mean time series and the time series from all voxels within the brain were estimated. The correlation values were then converted into z-scores using Fisher’s transform. Two-sample *t*-tests were performed to examine changes in connectivity between the patient group and the healthy control group. The resulting statistical maps were thresholded at *p* < 0.05 corrected for multiple comparison using FWE cluster level correction (FWEc) with cluster defining threshold set to *p* = 0.001. Seed-based connectivity analyses were performed using in-house Matlab scripts, while the two-sample *t*-tests were performed using SPM12.

### PET Study

THK5351 and PiB were prepared at the Cyclotron and Radioisotope section, Nagoya University. Radiosynthesis of Quinoline Derivatives THK5351 was prepared from the tosylate precursor (S)-2-(2-methylaminopyrid-5-yl)-6-[[2-(tetrahydro-2H-pyran-2-yloxy)-3-tosyloxy] propoxy] quinoline (THK5352) according to the previously described method for synthesizing using MPS-200 (Sumitomo Heavy Industries, Japan; Harada et al., 2016). Injectable solutions of THK5351 were prepared with a radiochemical purity of >95% and a specific activity of 113 ± 50 GBq/μmol. Radiosynthesis of the benzothiazole derivative PiB was synthesized using the one-step ^11^C- methyl triflate approach from the [N-methyl-11C]-6-OH-BTA-1 precursor (6-OH-BTA-0; Verdurand et al., 2008). PET imaging was performed using a Biograph 16 (Siemens Healthcare, Erlangen, Germany). After injecting 185 MBq of THK-5351 or 555 MBq of PiB, THK5351 PET images from 50 min to 60 min post-injection, PiB PET images from 50 min to 70 min post-injection were used for the following analysis.

### Regional Quantification of PiB PET Imaging

We used an automatic program, PMOD’s (version 3.7; PMOD Technologies Ltd., Zurich, Switzerland) PNEURO tool with brain volumes of interest (VOIs) by automatically leveraging the most likely localization of brain areas as encoded in the maximum probability atlas (N30R83) constructed by Hammers et al. (2003). The atlas was adjusted to the individual patient’s anatomy with a spatial normalization procedure, which was obtained from the T1-MR image. Standardized uptake values (SUV) images were acquired by normalizing tissue radioactivity concentration of PiB by injected dose and body weights. The regional SUV of all VOIs was divided by average of both side of cerebellar SUV to obtain the SUV ratio (SUVR) of each VOI. In PiB PET, if the average SUVRs of all neocortical areas, except for the medial temporal lobe, occipital lobe, pre- and post-central gyrus (global cortical SUVR), was larger than 1.5, we assessed the patients as “Aβ positive” (Jack et al., 2009; Bourgeat et al., 2010; Villemagne et al., 2011, 2013).

### PET (THK5351, PiB) Data Preprocessing and the Spatial Distribution Pattern Analysis

PET (THK5351, PiB) datasets were preprocessed using SPM 12. First, individual images were co-registered to the participants’ anatomical images. Using the deformation fields obtained in the segmentation stage, co-registered images were then normalized to the MNI space. After normalization, the images were resampled to a 2 × 2 × 2 mm^3^ voxel size and smoothed using an 8-mm FWHM isotropic 3D Gaussian filter. The preprocessed images were used in the subsequent analyses.

We performed SSM/PCA (Moeller and Strother, 1991; Alexander and Moeller, 1994; Spetsieris et al., 2013) on the obtained PET images to identify patterns that exhibited significant variance in THK5351/PiB concentration. SSM/PCA is a multivariate PCA-based algorithm that identifies major sources of variation in both patients’ and control group’s brain image data to reduce the complexity of multivariate data. To do this, the preprocessed PET images from all participants were transformed into a two-dimensional matrix where rows represent subjects and columns represent voxels. After applying a logarithmic transform to all elements in the matrix, the mean of each row (subject mean) was computed and subtracted from each row element. After this, the mean value of each column was also removed. Finally, the resulting matrix was analyzed using PCA to generate eigenimages and the associated subject-specific eigenimage scores which represented the similarity of each individual’s preprocessed THK5351/PiB concentration to the SSM/PCA-identified pattern. To delineate a specific AD-related topography, we limited the analysis to the set of principal components (PCs) that in aggregate accounted for the top 50% of subject × voxel variability, and for which each individual PC contributed at least 10% to the total variance in the scan data. (Niethammer et al., 2014). In the following analysis, we computed the correlation between component subject scores and ACE-R scores.

### Intrinsic Connectivity of Canonical Resting State Network and Distribution of THK5351 Retention

Using the preprocessed PET images, we also computed the similarity of the distribution between the THK5351 concentration and intrinsic connectivity within canonical RSNs (Myers et al., 2014). RSN masks were generated from the group RSNs obtained using group ICA (see “Canonical RSN Analysis,” section above). For each RSN and subject, the intrinsic connectivity values and THK5351 values were then extracted from all voxels within the RSN mask. Using these values, the similarity of the distribution between intrinsic connectivity and THK5351 was computed using Pearson’s correlation. Negative correlation indicates that voxels with high intrinsic connectivity have low THK5351 concentrations, while positive correlation means that voxels with high THK5351 concentrations have also high within network intrinsic connectivity. In other words, the more positive the correlation is, the more similar is the distribution. We tested for differences in the obtained similarity measure between the patient group and the healthy control group using a two-sample *t*-test.

### Comparative Study of the THK5351, Tau, MAO-B and Astrogliosis Findings of the Precuneus/Posterior Cingulate Cortex and Parahippocampus in Autopsy Cases With Alzheimer’s Disease

Two of the enrolled individuals were subjected to postmortem studies; an 87-year-old male (Case 1); and an 81-year-old female (Control 1). Pathological assays revealed that Case 1 had phosphorylated tau aggregations of Braak’s stage V, senile plaque of Consortium to Establish a Registry for AD (CERAD) C, and Aβ deposition of Thal’s Phase 5 (Montine et al., 2012); and the Control 1 did those corresponding to Braak’s stage II, CERAD B and Thal’s Phase 2. We did not find Lewy body pathology or TDP-43 inclusions among them. The pathological findings were compared to the relationship among tau, MAO-B and astrogliosis in parahippocampus and precuneus/posterior cingulate cortex (PCC). Neuropathological diagnostic analysis was performed on sections from a fixed left hemisphere by MY. The frozen tissue blocks were kept at −80°C in a deep freezer and sampled from the ~2-cm-thick coronal tissue slab of the parahippocampus and precuneus/PCC in the right hemisphere. Frozen tissues were sectioned from these blocks using a cryostat for autoradiography and immunohistochemistry. *In vitro* autoradiography was performed in the same protocol as previously described except for frozen sections (Harada et al., 2016, 2018). Immunohistochemistry was carried out using primary antibodies as follows; anti-phosphorylated tau (AT8, anti-mouse monoclonal, 1:200, Innogenetics, Ghent, Belgium), anti-Aβ (6F/3D, anti-mouse monoclonal, 1:50, Dako, Glostrup, Denmark), anti-GFAP (6F2, anti-mouse polyclonal, 1:100, Dako, Glostrup, Denmark) and anti-MAO-B (anti-rabbit polyclonal, 1:200, Sigma-Aldrich, St. Louis, MO, USA). The specimens of immunofluorescence were observed using Nikon Eclipse microscope (Tokyo, Japan).

### Statistical Analysis

Results of groups were expressed as mean ± SD. Clinical backgrounds were compared using a non-parametric test (Mann-Whitney test or chi-squared test). We examined Spearman’s rank correlation coefficient to reveal the correlation between the ACE-R scores and component subject scores of the spatial distributions of THK5351/PiB covariation pattern. All statistical tests were two-tailed. The statistical significance threshold was set at *p* < 0.05. Statistical analyses were performed using the Statistical Package for the Social Sciences (SPSS) version 24 (SPSS Inc., Chicago, IL, USA).

## Results

### Patients’ Characteristics

There were no significant differences in age at examination, male-to-female ratio, and education levels between early AD and healthy controls. In early AD, 15 had a CDR score of 0.5, and eight had a score of 1.0. Significantly different scores of MMSE, ADAS-cog-j, logical memory II and ACE-R scores were found between early AD and healthy controls (Table 1).

**Table 1:** Patients’ characteristics.

	Healthy control (HC)	Early AD	*p* value
Number	24	23	N.A.
Age at examination	65.4 ± 7.3	68.6 ± 7.8	N.S.^a^
Male:Female	8:16	4:19	N.S.^b^
Education	14.1 ± 2.0	13.4 ± 1.9	N.S.^a^
Age at onset	NA	66.1 ± 7.9	N.A.
Disease duration (year)	NA	2.5 ± 1.4	N.A.
CDR	0.0 ± 0.0	0.67 ± 0.24	<0.001^a^
CDR-SB	0.0 ± 0.0	2.9 ± 1.2	<0.001^a^
MMSE	29.4 ± 1.0	23.6 ± 2.8	<0.001^a^
ADAS-cog-j	3.0 ± 1.4	12.6 ± 5.3	<0.001^a^
Logical memory II	21.1 ± 6.7	1.0 ± 1.7	<0.001^a^
ACE-R	97.9 ± 2.4	75.1 ± 9.0	<0.001^a^
Aβ global SUVR	1.18 ± 0.08	1.93 ± 0.25	<0.001^a^

*Data are shown as mean ± standard deviation (SD). ^a^Healthy control vs. early AD using Mann-Whitney, ^b^Healthy control vs. early AD using chi-square*. *MMSE, mini mental state examination; CDR, clinical dementia rating; CDR-SB, Clinical Dementia Rating Scale Sum of Boxes; ADS-cog-j, Alzheimer’s Disease Assessment Scale-Cognitive-Japanese; ACE-R, Addenbrooke’s Cognitive Examination-Revised; Aβ, amyloid β; SUVR, Standard uptake value ratio; AD, Alzheimer’s disease; N.S., not significant; N.A., not applicable*.

### Voxel-Based Morphometry Findings

**Figure 1:**
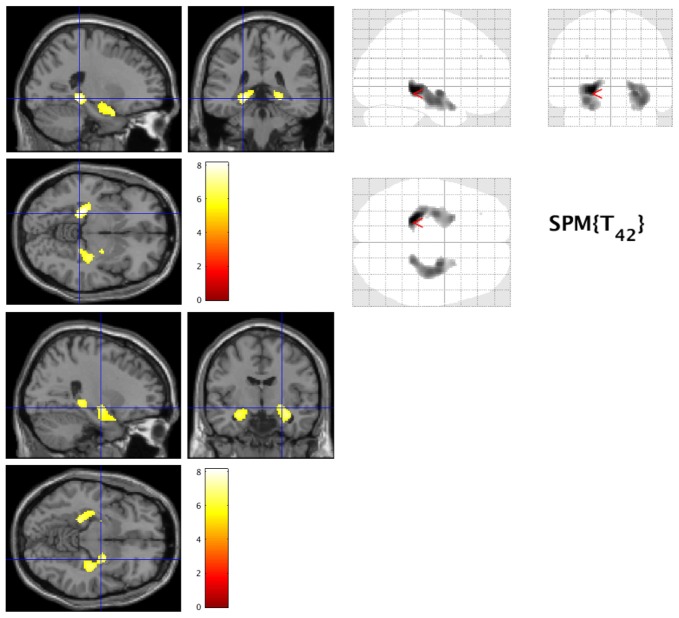
Decreased gray matter volume in early Alzheimer’s disease (AD). The hot spots indicate sites where gray matter’s volume significantly decreased in AD (family-wise error cluster level correction (FWEc) at *p* < 0.05).

Patients with early AD showed significantly decreased GM volume in the bilateral lingual gyrus (Brodmann area (BA) 30) and parahippocampal gyrus (BA30) compared to healthy controls (FWE at *p* < 0.05, Figure 1).

### Spatial Distributions of THK5351 Using Scaled Subprofile Modeling/Principal Component Analysis (SSM/PCA)

**Figure 2:**
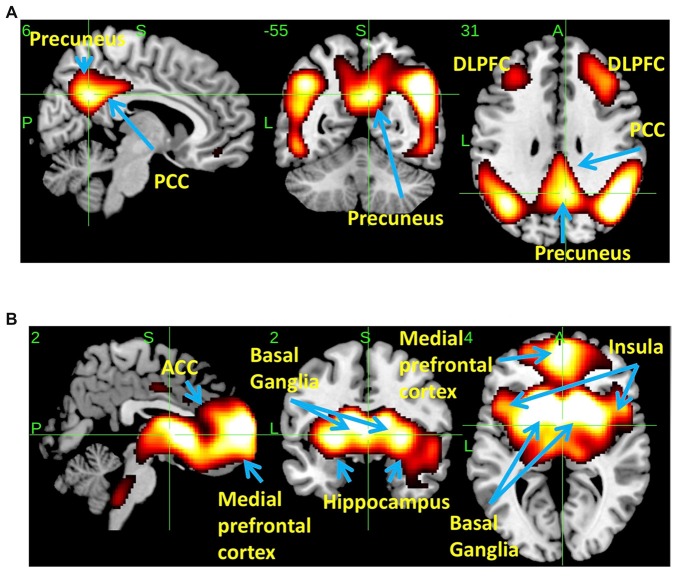
Spatial distributions of THK5351 covariation pattern using scaled subprofile modeling/principal component analysis (SSM/PCA). AD-related THK5351 distribution pattern (ADRTP; **A**): SSM/PCA identified a principal component 1 (PC1), which indicates AD-related THK5351 covariation pattern (ADRTP). The hot areas represent PC1. Common distribution pattern of THK5351 between healthy control (HC) and AD **(B)**: SSM/PCA identified PC2, which indicates that the accumulation sites common to early AD patients and HC. The hot areas represent PC2.

**Figure 3:**
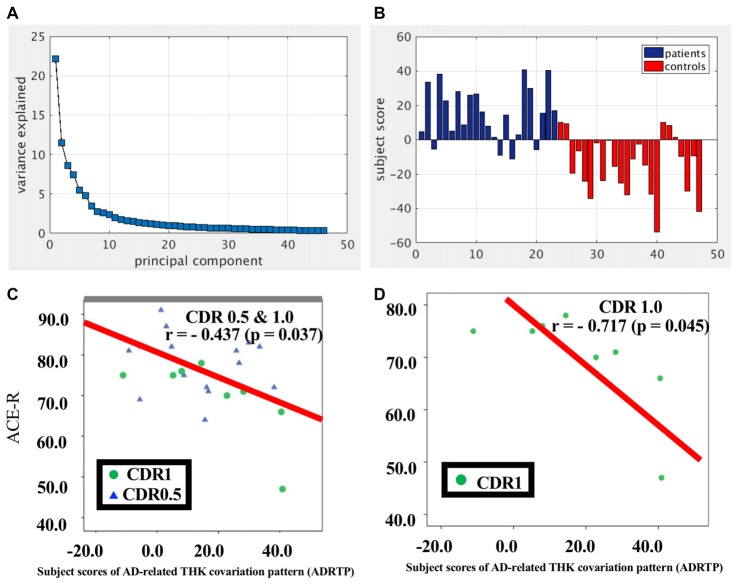
PC1 and subject score, ADRTP. Panel **(A)** shows ADRTP. This figure indicates the ratio of each PC with the total subject voxel variance of data. Panel **(B)** shows subject score relative to PC1 which showed 82.6% sensitivity and 79.1% specificity in discriminating AD from HC. Panel **(C)** shows the relationship between the Addenbrooke’s Cognitive Examination Revised (ACE-R) and the ADRTP subject scores of early AD patients, Clinical Dementia Rating 1.0 (CDR 1.0) and CDR 0.5. Blue triangles indicate CDR0.5, and green circles indicate CDR1.0. Panel **(D)** represents the relationship between ACE-R and the ADRTP subject scores of early AD patients, CDR1.0. Green circles indicate CDR1.0.

SSM/PCA of the THK5351PET imaging data identified a significant spatial THK5351 pattern corresponding to principal component 1 (PC1) (Figure 2A), which accounted for 23.6% of the total subject voxel variance of the data (Figure 3A) and had 82.6% sensitivity and 79.1% specificity in discriminating AD from healthy controls (Figure 3B). Thus, we called this distribution pattern (PC1) as AD-related THK5351 distribution pattern (ADRTP) and its PC score as ADRTP score. The ADRTP was composed of three major clusters (Table 2). Clusters 1 and 2 of ADRTP mainly included bilateral inferior, middle and superior frontal gyrus (BA 6, 8, 9, 10; cluster size >100). Cluster 3 of ADTRP included the inferior parietal lobule (BA 40), precuneus (BA7, 19), posterior cingulate cortex (BA31), inferior (BA20, 21), middle (BA39), and superior temporal gyrus (BA22), and fusiform gyrus (BA37; cluster size >100; Table 2). Among the ADRTP-related regions, the most prominent areas were the precuneus and PCC (Cluster 3), followed by the lateral middle and superior frontal gyri, which is well known as the dorsolateral prefrontal cortex (DLPFC; Figure 2A, Table 2) corresponding to Braak IV and V. The correlation between subject scores of ADRTP and ACE-R scores was significant in all participants (*r* = −0.68, *p* = 1.9 × 10^−7^; Figure 3C), in only early AD patients with a CDR score of 0.5 and 1.0 (*r* = −0.44, *p* = 0.037; Figure 3C), and in early AD with a CDR score of 1.0 (*r* = −0.72, *p* = 0.045; Figure 3D), but not in healthy controls (*r* = 0.10, *p* = 0.7).

**Table 2:** Location of AD-related THK5351 distribution pattern (PC1).

Area	Number of voxels	Side	BA	Location of peak within area		
				*x*	*y*	*z*
**Cluster 1**
Middle frontal gyrus	186	Left	BA9	−32	30	38
Superior frontal gyrus	123	Left	BA8	−34	24	44
**Cluster 2**
Middle frontal gyrus	363	Right	BA9	34	26	38
Superior frontal gyrus	334	Right	BA8	34	16	48
Medial frontal gyrus	249	Right	BA10	28	44	26
Inferior frontal gyrus	229	Right	BA6	32	12	48
**Cluster 3**
Inferior parietal lobule	1227	Bilateral	BA40	48	−54	38
Precuneus	1162	Bilateral	BA7	4	−58	30
Inferior temporal gyrus	834	Bilateral	BA20	56	−30	−20
Inferior temporal gyrus	795	Bilateral	BA21	58	−26	−16
Middle temporal gyrus	760	Bilateral	BA39	46	−62	28
Precuneus	716	Bilateral	BA7	4	−54	30
Fusiform gyrus	354	Bilateral	BA37	54	−40	−20
Precuneus	236	Bilateral	BA19	34	−68	38
Superior temporal gyrus	195	Bilateral	BA22	50	−60	14

*BA, Brodman area*.

PC2 (Figure 2B), the second most important PC accounting for 11.8% of the total subject voxel variance, included the bilateral basal ganglia, hippocampus, medial prefrontal cortex and anterior cingulate cortex—regions where tau distribution and increased MAO-B was commonly observed in healthy aged controls (Lemoine et al., 2017; Ng et al., 2017). Scores based on PC2 had 70.8% sensitivity and 52.2% specificity in discriminating healthy controls from AD. PC2 scores did not show any significant correlation with ACE-R and CDR.

### Spatial Distributions of Amyloid β Pattern Using SSM/PCA

We also identified significant spatial Aβ covariation patterns (AD related Aβ distribution pattern, ADRAP; Supplementary Figure S1A) that showed an analogous distribution of ADRTP and accounted for greater than 50% of the data’s variance (Supplementary Figure S1B). However, ADRAP was more widespread in the medial frontal gyrus (BA6, 9, 10, 11), precuneus (BA7), supramarginal gyrus (BA40), middle temporal gyrus (BA21, 39, 46), superior temporal gyrus (BA13, 22, 42), anterior cingulate cortex (BA32), insula (BA47), and posterior cingulate cortex (BA23). ADRAP had 100% sensitivity and 100% specificity in discriminating AD from healthy controls. This result was quite reasonable because we included PiB positive AD patients and PiB negative healthy controls based on global SUVR higher than 1.5 or not (Supplementary Figure S1C). However, the correlations between subjects’ ADRAP scores and ACE-R scores were not significant in early AD patients (*r* = −0.04, *p* = 0.8) irrespective of CDR score (0.5 and 1.0, *r* = 0.04; 1.0, *r* = −0.03), or in healthy controls (*r* = 0.06, *p* = 0.8).

### Relationship Between Spatial Distribution of THK5351 Retention and Canonical Resting State Networks

**Figure 4:**
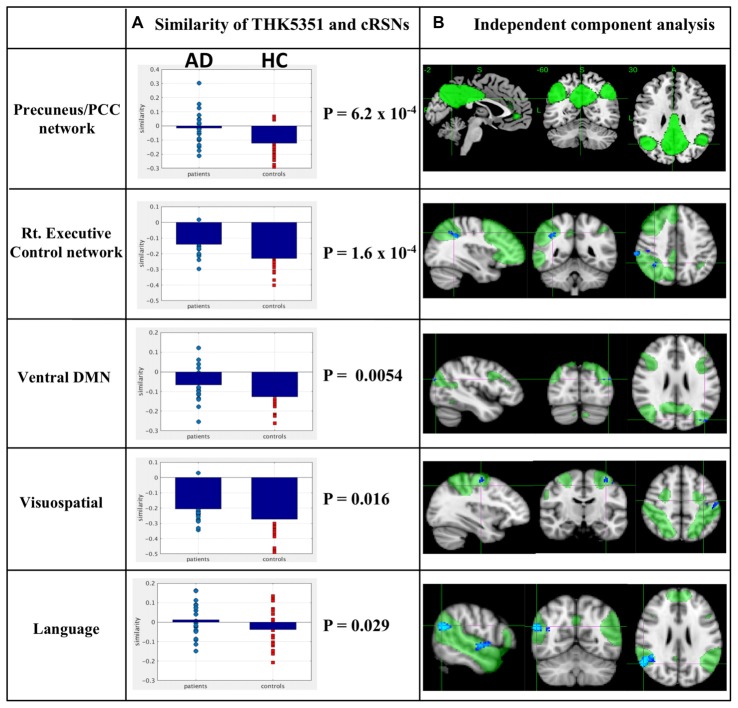
Relationship between THK5351 retention and canonical resting state networks (RSNs). Panel **(A)** shows the similarity of the THK5351 retention pattern and each canonical RSN. Panel **(B)** is the result of independent component analysis (ICA). The green areas indicate each canonical network, and the blue areas indicate decreased connectivity, as identified by ICA of resting-state functional MRI data (FWE at *p* < 0.05).

The estimated similarity values of the distribution between THK5351 concentration and intrinsic functional connectivity values within canonical RSNs computed as the correlation between the two variables were mostly negative in healthy controls (Figure 4). This indicates that, in healthy controls, voxels with higher intrinsic connectivity within the network is associated with lower THK5351 concentration. However, in the patient group, a shift towards a more positive association, i.e., higher connectivity, higher THK5351 concentration, was observed and was significant in some canonical RSNs. Two-sample *t*-test comparisons of the similarity between the spatial distribution patterns of THK5351 retention and within-network intrinsic functional connectivity showed significant difference in the precuneus/PCC network (*p* = 6.2 × 10^−4^) and right executive control network (*p* = 1.6 × 10^−4^), followed by ventral DMN (*p* = 0.0054), visuospatial network (*p* = 0.016) and language network (*p* = 0.029). The results of ICA and dual regression analyses also showed decreased connectivity within the right executive control network, ventral DMN, visuospatial network and language network (FWE at *p* < 0.05).

### Seed-Based Analysis of Functional Connectivity of Highly THK5351-Retaining Regions

**Figure 5:**
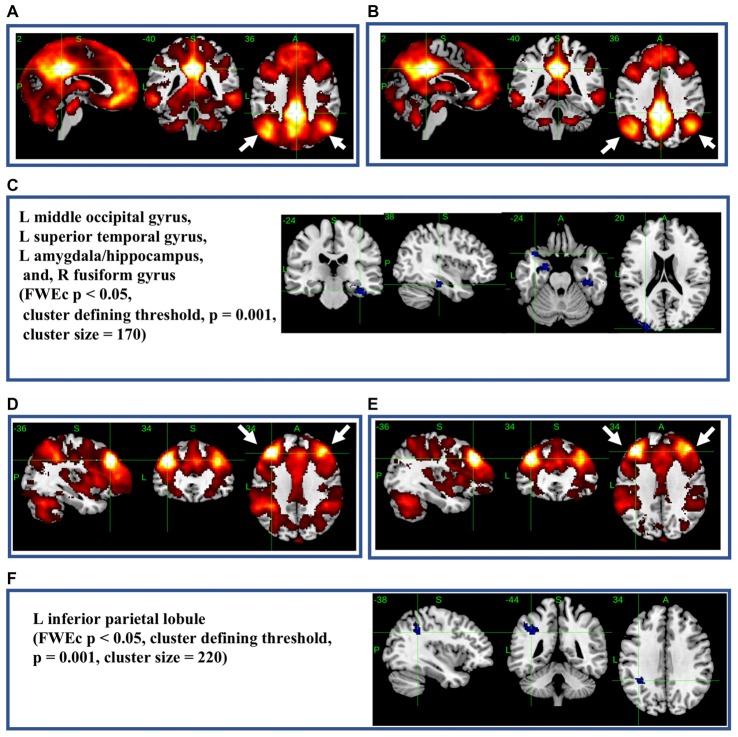
Seed-based connectivity of the precuneus/posterior cingulate cortex (PCC) and dorsolateral prefrontal cortex (DLPFC). This figure shows seed-based connectivity analysis from ROIs located in the precuneus/PCC **(A–C)**, and DLPFC **(D–F)**. **(A,D)** HC **(B,E)** early AD; **(C)** intrinsic connectivity of precuneus/PCC significantly decreased to the hot areas (FWEc *p* < 0.05, cluster defining threshold, *p* = 0.001, cluster size = 170). **(F)** Intrinsic connectivity of DLPFC significantly decreased to the hot areas (FWEc *p* < 0.05, cluster defining threshold, *p* = 0.001, cluster size = 220).

The most significant difference in THK5351 retention between early AD and healthy controls in the ADRTP was observed in the bilateral precuneus/PCC and the left DLPFC. Thus, we performed seed-based connectivity analysis using two ROIs as the seed regions. The mean connectivities of the precuneus/PCC and the DLPFC in healthy controls is shown in Figures 5A,D, while those in early AD is shown in Figures 5B,E. The intrinsic connectivity of precuneus/PCC significantly decreased in the left middle occipital gyrus, left superior temporal gyrus, left amygdala/hippocampus and right fusiform gyrus (FWEc *p* < 0.05, cluster defining threshold, *p* = 0.001, cluster size = 170; Table 3, Figure 5C). That of left DLPFC decreased to the left inferior parietal lobule (FWEc *p* < 0.05, cluster defining threshold, *p* = 0.001, cluster size = 220; Table 3, Figure 5F).

**Table 3:** Seed based connectivity analysis.

	Cluster			BA	Side	MNI coordinates		
	PFWE-corr	K(E)	p-uncorrected			*x*	*y*	*z*
**Left PCC area**
Middle occipital gyrus	0.002	322	0.000	19	L	−28	−96	20
Superior temporal gyrus	0.018	214	0.001	38	L	38	−24	22
Amygdala	0.035	183	0.002		L	−24	−2	−22
Fusiform gyrus	0.047	170	0.003	2	R	−38	18	−24
**Left DLPFC area**
Superior parietal lobule	21	220	0.001	7	L	−38	−44	34

*FWE, Family wised error; K(E), minimum cluster extent; BA, Broadman area*.

### Comparative Study Among THK5351, Tau, MAO-B and Astrogliosis Findings of the Precuneus/PCC in Autopsy Cases With Alzheimer’s Disease

**Figure 6:**
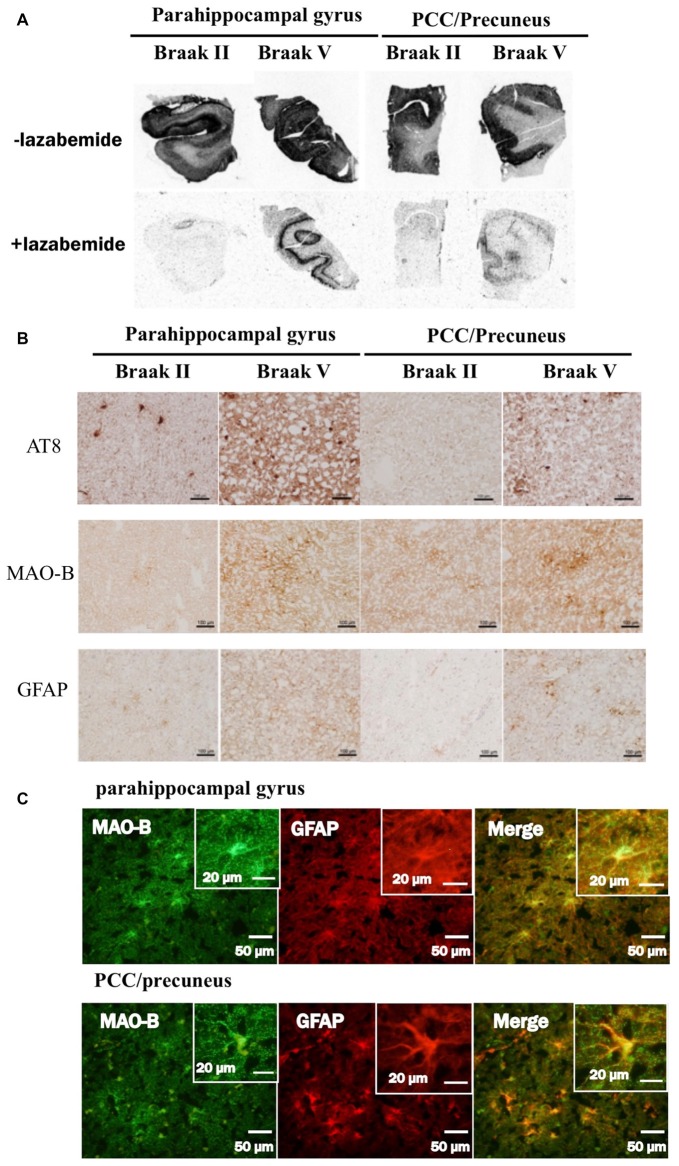
Comparative study among THK5351, tau, monoamine oxidase-B (MAO-B) and astrogliosis findings of the precuneus/posterior cingulate gyrus (PCC) and parahippocampal gyrus in autopsy cases with AD. Panel **(A)** shows the results of ^3^H-THK5351 autoradiography. *In vitro* autoradiogram of ^3^H-THK5351 in brain sections of the parahippocampal gyrus and precuneus/PCC from an 81-year-old control (Braak II) and an 87-year-old AD patient (Braak V) in the absence and presence of the MAO-B inhibitor lazabemide. ^3^H-THK5351 bindings in the parahippocampal gyrus and precuneus/PCC was more evident in a patient with Braak’s stage V (Case 1) than in a healthy control with Braak’s stage II (Control 1). After treatment with lazabemide (+lazabemide) as a MAO-B inhibitor, ^3^H-THK5351 bindings remained detectable in the parahippocampus and precuneus/PCC in Case 1 with Braak’s stage V but was scarce in Control 1 with Braak’s stage II. Panel **(B)** demonstrates the results of immunostaining of tau, MAO-B, and glial fibrillary acidic protein (GFAP). Anti-tau antibody (AT8), anti-MAO-B antibody and GFAP immunohistochemistry revealed marked immunostaining in an AD case, compared to a healthy control. Tau, MAO-B and GFAP accumulation were more evident in Braak’s stage V than in Braak’s stage II patients. Panel **(C)** is the result of double immunofluorescence for GFAP and MAO-B. Tissue sections double stained with anti-MAO-B antibody and anti-GFAP antibody in a Braak’s stage V parahippocampal gyrus, and the PCC/precuneus. GFAP and MAO-B were colocalized in the astrocyte. Anti-GFAP immunohistochemistry diffusely labeled the cytoplasm and processed of astrocytes, which contained MAO-B immunopositive granules.

In autoradiograhy, ^3^H-THK5351 bindings in the parahippocampal gyrus and precuneus/PCC was more evident in a patient with Braak’s stage V (Case 1) than in a healthy control with stage II (Control 1, Figures 6A,B). After treatment with lazabemide as a MAO-B inhibitor, ^3^H-THK5351 bindings remained detectable in the parahippocampus and precuneus/PCC in Case 1 with Braak’s stage V, but was scarce in Control 1 with Braak’s stage II (Figure 6A). Immunostaining showed that tau, MAO-B, and GFAP accumulation were more severe in Case 1 with Braak’s stage V than in Control 1 with Braak’s stage II (Figure 6B). Double immunofluorescence revealed co-localization of GFAP and MAO-B within in the astrocyte (Figure 6C). Anti-GFAP immunohistochemistry diffusely labeled the cytoplasm and processes of astrocytes, which contained MAO-B immunopositive granules.

## Discussion

### The Significance of Precuneus/PCC Involvement for the Development of Alzheimer’s Disease Symptoms

This is the first study to demonstrate ADRTP in early AD using SSM/PCA, a non-biased data-driven approach. The characteristics of AD features were as follows: (1) the most prominent AD-specific THK5351 retention areas in the ADRTP were the precuneus/PCC followed by the DLPFC; (2) the subject ADRTP scores were significantly correlated with the cognitive scores; (3) the significant correlation of distribution pattern of THK5351 retention to the intrinsic connectivity within canonical RSNs was observed in the precuneus/PCC networks; (4) seed-based connectivity analysis of the precuneus/PCC showed significantly decreased connectivity to widespread regions associated with cognitive function; and (5) pathologically, THK5351 retention was associated with tau deposition, astrogliosis and MAO-B and was more evident in the precuneus/PCC with Braak’s stage V (Alzheimer type dementia) than the precuneus/PCC with Braak’s stage II (healthy control). These findings support the view that tau retention and astrogliosis/neurodegeneration in the precuneus/PCC will play an important role in determining the dementia state in early AD.

The ADRTP (PC1) discriminated the AD-specific THK5351 deposition pattern and indicated the potential involvement of canonical brain networks in AD. In addition, the subject score indicating the similarity to the ADRTP (PC1), showed a significant association with cognitive function in early AD patients, particularly patients with CDR equal to 1.0. Thus, ADRTP could provide an important measure for the diagnosis of early AD. Indeed, the ADRTP subject score showed good sensitivity and specificity in differentiating early AD patients from healthy controls.

The precuneus network is part of the classical DMN along with the dorsal DMN and ventral DMN. In addition, both the precuneus and PCC show high levels of metabolism, are vascular boundaries, have widespread connections to other brain regions serving as brain network hubs, and key nodes in the classical DMN (Cavanna and Trimble, 2006; Zhang and Li, 2012; Leech and Sharp, 2014; Utevsky et al., 2014). Pathologically, the precuneus/PCC is involved in Braak’s stage IV, leading to the appearance of initial clinical symptoms, and Braak’s stage V, which is associated with full development of AD. As one of the most important brain network hub regions, tau retention and astrogliosis/neurodegeneration extending to the precuneus/PCC could therefore lead to widespread disruption of brain networks responsible for the maintenance of normal cognitive function, which in turn could result in the manifestation of dementia in AD. Indeed, in our study, functional connections from the precuneus/PCC were significantly altered over widespread regions in the brain. More recently, Hoenig et al. (2018) also demonstrated the significant overlap between distribution of AV1451PET (tau PET probe) and canonical networks especially, ventral and dorsal DMN assessed by ICA.

Aside from the precuneus/PCC, ADRTP also showed the DLPFC which is brain network hub regions (Bullmore and Sporns, 2012) as the second most prominent region involved in early AD. These prefrontal cortices can also show the differences between AD and healthy controls (Stam, 2014). These results further indicate that THK5351 retention in the brain hub regions would be closely associated with the development of dementia in AD. Dynamic network changes would occur in the DLPFC and precuneus/PCC during disease progression from healthy aging to MCI or AD (Binnewijzend et al., 2012; Brier et al., 2012; Adriaanse et al., 2014), which would also play an important compensatory role (Bozzali et al., 2015; Wang et al., 2015; Elman et al., 2016; Serra et al., 2016).

### PiB Retention (Amyloid β Deposition) and Resting State Networks

In this study, we also identified ADRAP using SSM/PCA. The ADRAP subject score did not show any significant correlation with ACE-R in AD (*r* = −0.04, *p* = 0.8) in contrast to ADRTP (Figure 4D), confirming previous reports (Fripp et al., 2008) and supporting the view that PiB deposition is not independently correlated to the development of dementia (Rowe et al., 2007; Jack et al., 2009; Canter et al., 2016). Aβ retention topography exhibits a high degree of overlap with the DMN (Buckner et al., 2008; Canter et al., 2016) and other intrinsic network disruptions in AD (Sheline et al., 2010; Lim et al., 2014; Busche et al., 2015). While it is widely accepted that there is a significant discrepancy between the Aβ pathological severity of AD and its clinical phenotype (Jack et al., 2009; Canter et al., 2016). Thus, our results on the lack of relationship between PiB retention and the decline of cognitive function are also consistent with previous reports (Jack et al., 2009; Villemagne et al., 2011) and may support the view that tau deposition and astrogliosis/neurodegeneration, rather than Aβ pathology, have a close relationship with cognitive decline, predominantly memory impairment, in AD.

### Relationship Among MAO-B, Astrogliosis and Tau

Recently, regional *in vivo* THK5351 retention was reported to be significantly correlated with the density of tau aggregates in the neocortex and MAO-B in whole brain (Ng et al., 2017; Harada et al., 2018) as compared to AV-1451 (Jang et al., 2018). Furthermore, a significant association was observed between the density of tau aggregates, MAO-B and GFAP, suggesting that neocortical tau is strongly correlated with the formation of reactive astrocytes. In this study, we confirmed that increased MAO-B and astrogliosis co-localized according to double immunostaining in the involved regions. Astrogliosis is linked to neurodegeneration/neuronal loss in many neurodegenerative disorders, although it is still debatable whether astrogliosis or neurodegeneration is the earlier event. The fact that THK5351 binds to the MAO-B related to astrogliosis (Ng et al., 2017; Harada et al., 2018) supports the view that THK5351 imaging has potential for visualizing tau pathology, astrogliosis and neurodegeneration. Significant negative correlation between THK5351 retention and glucose hypometabolism in AD were also reported (Kang et al., 2017). In addition, SSM/PCA clearly demonstrated the discrimination ADRTP (PC1) and non-specific THK5351 retention (PC2) in this study. Thus, the spatial THK5351 retention pattern demonstrated by ADRTP could reflect the AD pathology rather than the restricted tau pathology. However, further clinical, radiological, and pathological studies with a larger number of patients will be needed to clarify this issue.

Tau aggregation and astrogliosis are two quite distinct biological mechanisms, both of which are the critical features of pathogenic mechanisms for AD development, and also both are closely linked in AD pathologic legions. Unfortunately, THK5351 as state of the art cannot distinguish between tau accumulation and astrogliosis. However, we speculated that THK5351 could detect both pathologic processes simultaneously and can be a marker for these process in AD. Future development of PET tracers which have improved binding selectivity and pharmacokinetics to tau and MAO-B/astrogliosis will help not only for better understanding the underlying mechanism of AD but also for clinical trials targeting astrogliosis (Okamura et al., 2018).

### Relationship Between Hubs of the Brain Networks and AD Pathology

Within large-scale brain networks, hubs have a remarkably high number of connections through which they integrate the functions of distant networks. More energy probably will be needed to maintain hub function as compared to other regions (Achard and Bullmore, 2007; He et al., 2009). The sustained metabolic activation of the brain’s DMN is thought to render the system vulnerable to AD. Recent studies demonstrated that enhanced neuronal activity could increase the release and transfer of tau and Aβ *in vitro* and exacerbate tau Aβ pathology *in vivo* (Wu et al., 2016), suggesting that increasing hub activities may be associated with acceleration of Aβ and tau accumulations. Thus, one hypothesis could be derived based on the balance between intrinsic vulnerabilities with increasing activities of neuronal subpopulations to stressors, and specific disease-related misfolding proteins may determine the neuronal and network involvements (Saxena and Caroni, 2011). Although recent developments of network science have demonstrated the overload and failure of hubs as a conceivable final common pathway in neurodegenerative disorders (Stam, 2014), further study will be needed to ascertain the role of hubs in both compensating cognitive function and accelerating abnormal Aβ and tau accumulations.

## Author Contributions

The data of this study were acquired by TY, HW, HY, MM, KI, AO, RO, KazuyaK, KH, SM, KatsuhikoK, RH and MY. TY, HW, EB and GS analyzed the data, interpreted the results and drafted this manuscript. YR, SI, SM, MK, SN, RH, NO, KY and MY revised this manuscript critically for important intellectual content. All authors approved the final version of this manuscript.

## Conflict of Interest Statement

The authors declare that the research was conducted in the absence of any commercial or financial relationships that could be construed as a potential conflict of interest.

## Footnotes

^1^http://www.fil.ion.ucl.ac.uk/spm/software/spm12/

^2^http://www.fmrib.ox.ac.uk/fsl

## Supplementary Material

FIGURE S1 Spatial distributions of the amyloid β (PiB) covariation pattern using SSM/PCA. Panel **(A)** shows the Alzheimer’s disease-related Aβ distribution pattern (ADRAP). SSM/PCA identified an Alzheimer’s disease-related Aβ covariation pattern (ADRAP). The hot areas represent principal component 1 of ADRAP. Panel **(B)** is the Alzheimer’s disease-related PiB distribution pattern. This figure indicates the ratio of each principal component with the total subject voxel variance of data. Panel **(C)** is Subject score relative to principal component 1 of Alzheimer’s disease-related amyloid β (PiB) covariation pattern.

## Abbreviations

ACE-R: the Addenbrooke’s Cognitive Examination Revised
ADAS-cog-J: the Alzheimer’s Disease Assessment Scale-Cognitive-Japanese
ADRAP: Alzheimer’s disease related amyloid βdistribution pattern
ADRTP: Alzheimer’s disease-related THK5351 distribution pattern
BA: brodmann area
CDR: Clinical Dementia Rating
CERAD: Consortium to Establish a Registry for Alzheimer’s Disease
CSF: cerebrospinal fluid
DLPFC: dorsolateral prefrontal cortex
DMN: default mode network
FOV: field of view
FWE: family-wise error
FWEc: FWE cluster level correction
FWHM: full-width-at-half-maximum
GFAP: glial fibrillary acidic protein
GM: gray matter
ICA: independent component analysis
MAO-B: monoamine oxidase-B
MCI: minimal cognitive impairment
MMSE: the mini mental state examination
MNI: Montreal Neurological Institute
PCA: principal component analysis
PCC: posterior cingulate cortex
PiB: ^11^C-Pittsburgh compound B
rsfMRI: resting state functional MRI
RSN: resting state network
SPM: statistical parametric mapping
SSM/PCA: scaled subprofile modeling/principal component analysis
SUV: standardized uptake values
SUVR: SUV ratio
TE: echo time
THK5351: ^18^F-THK5351
TR: repetition time
WM: white matter.

## References

[B1] Achard S. Bullmore E. (2007). Efficiency and cost of economical brain functional networks. PLoS Comput. Biol. 3:e17. 10.1371/journal.pcbi.0030017 17274684 PMC1794324

[B3] Adriaanse S. M. Binnewijzend M. A. Ossenkoppele R. Tijms B. M. van der Flier W. M. Koene T.. (2014). Widespread disruption of functional brain organization in early-onset alzheimer’s disease. PLoS One 9:e102995. 10.1371/journal.pone.0102995 25080229 PMC4117463

[B4] Agosta F. Pievani M. Geroldi C. Copetti M. Frisoni G. B. Filippi M. (2012). Resting state fMRI in Alzheimer’s disease: beyond the default mode network. Neurobiol. Aging 33, 1564–1578. 10.1016/j.neurobiolaging.2011.06.007 21813210

[B5] Alexander G. E. Moeller J. R. (1994). Application of the scaled subprofile model to functional imaging in neuropsychiatric disorders: a principal component approach to modeling brain function in disease. Hum. Brain Mapp. 2, 79–94. 10.1002/hbm.460020108

[B6] Ashburner J. (2007). A fast diffeomorphic image registration algorithm. Neuroimage 38, 95–113. 10.1016/j.neuroimage.2007.07.007 17761438

[B7] Binnewijzend M. A. Schoonheim M. M. Sanz-Arigita E. Wink A. M. van der Flier W. M. Tolboom N.. (2012). Resting-state fMRI changes in Alzheimer’s disease and mild cognitive impairment. Neurobiol. Aging 33, 2018–2028. 10.1016/j.neurobiolaging.2011.07.003 21862179

[B8] Bourgeat P. Chételat G. Villemagne V. L. Fripp J. Raniga P. Pike K.. (2010). β-Amyloid burden in the temporal neocortex is related to hippocampal atrophy in elderly subjects without dementia. Neurology 74, 121–127. 10.1212/WNL.0b013e3181c918b5 20065247

[B9] Bozzali M. Dowling C. Serra L. Spanò B. Torso M. Marra C.. (2015). The impact of cognitive reserve on brain functional connectivity in Alzheimer’s disease. J. Alzheimers Dis. 44, 243–250. 10.3233/JAD-141824 25201783

[B10] Brier M. R. Thomas J. B. Snyder A. Z. Benzinger T. L. Zhang D. Raichle M. E.. (2012). Loss of intranetwork and internetwork resting state functional connections with Alzheimer’s disease progression. J. Neurosci. 32, 8890–8899. 10.1523/JNEUROSCI.5698-11.2012 22745490 PMC3458508

[B11] Buckner R. L. Andrews-Hanna J. R. Schacter D. L. (2008). The brain’s default network: anatomy, function and relevance to disease. Ann. N Y Acad. Sci. 1124, 1–38. 10.1196/annals.1440.011 18400922

[B12] Bullmore E. Sporns O. (2012). The economy of brain network organization (2012). Nat. Rev. Neurosci. 13, 336–349. 10.1038/nrn3214 22498897

[B13] Busche M. A. Kekuš M. Adelsberger H. Noda T. Forstl H. Nelken I.. (2015). Rescue of long-range circuit dysfunction in Alzheimer’s disease models. Nat. Neurosci. 18, 1623–1630. 10.1038/nn.4137 26457554

[B14] Canter R. G. Penney J. Tsai L. H. (2016). The road to restoring neural circuits for the treatment of Alzheimer’s disease. Nature 539, 187–196. 10.1038/nature20412 27830780

[B15] Cavanna A. E. Trimble M. R. (2006). The precuneus: a review of its functional anatomy and behavioural correlates. Brain 129, 564–583. 10.1093/brain/awl004 16399806

[B16] Damoiseaux J. S. Prater K. E. Miller B. L. Greicius M. D. (2012). Functional connectivity tracks clinical deterioration in Alzheimer’s disease. Neurobiol. Aging 33, 828.e19–828.e30. 10.1016/j.neurobiolaging.2011.06.024 21840627 PMC3218226

[B18] Ekblom J. Jossan S. S. Bergström M. Oreland L. Walum E. Aquilonius S. M. (1993). Monoamine oxidase-B in astrocytes. Glia 8, 122–132. 10.1002/glia.440080208 8406673

[B19] Elman J. A. Madison C. M. Baker S. L. Vogel J. W. Marks S. M. Crowley S.. (2016). Effects of β-amyloid on resting state functional connectivity within and between networks reflect known patterns of regional vulnerability. Cereb. Cortex 26, 695–707. 10.1093/cercor/bhu259 25405944 PMC4712800

[B20] Fazekas F. Kleinert R. Offenbacher H. Fazekas F. Payer F. Schmidt R.. (1991). The morphologic correlate of incidental punctate white matter hyperintensities on MR images. Am. J. Neuroradiol. 12, 915–921. 1950921 PMC8333504

[B21] Filippini N. MacIntosh B. J. Hough M. G. Goodwin G. M. Frisoni G. B. Smith S. M.. (2009). Distinct patterns of brain activity in young carriers of the *APOE*-ε4 allele. Proc. Natl. Acad. Sci. U S A 106, 7209–7214. 10.1073/pnas.0811879106 19357304 PMC2678478

[B22] Fripp J. Bourgeat P. Acosta O. Raniga P. Modat M. Pike K. E.. (2008). Appearance modeling of ^11^C PiB PET images: characterizing amyloid deposition in Alzheimer’s disease, mild cognitive impairment and healthy aging. Neuroimage 43, 430–439. 10.1016/j.neuroimage.2008.07.053 18789389

[B23] Greicius M. D. Srivastava G. Reiss A. L. Menon V. (2004). Default-mode network activity distinguishes Alzheimer’s disease from healthy aging: evidence from functional MRI. Proc. Natl. Acad. Sci. U S A 101, 4637–4642. 10.1073/pnas.0308627101 15070770 PMC384799

[B24] Hammers A. Allom R. Koepp M. J. Free S. L. Myers R. Lemieux L.. (2003). Three-dimensional maximum probability atlas of the human brain, with particular reference to the temporal lobe. Hum. Brain Mapp. 19, 224–247. 10.1002/hbm.10123 12874777 PMC6871794

[B25] Harada R. Ishiki A. Kai H. Sato N. Furukawa K. Furumoto S.. (2018). Correlations of ^18^F-THK5351 PET with postmortem burden of Tau and astrogliosis in Alzheimer disease. J. Nucl. Med. 59, 671–674. 10.2967/jnumed.117.197426 28864633

[B26] Harada R. Okamura N. Furumoto S. Furukawa K. Ishiki A. Tomita N.. (2016). ^18^F-THK5351: a novel PET radiotracer for imaging neurofibrillary pathology in Alzheimer disease. J. Nucl. Med. 57, 208–214. 10.2967/jnumed.115.164848 26541774

[B27] He Y. Wang J. Wang L. Chen Z. J. Yan C. Yang H.. (2009). Uncovering intrinsic modular organization of spontaneous brain activity in humans. PLoS One 4:e5226. 10.1371/journal.pone.0005226 19381298 PMC2668183

[B29] Hoenig M. C. Bischof G. N. Seemiller J. Hammes J. Kukolja J. Onur O. A.. (2018). Networks of tau distribution in Alzheimer’s disease. Brain 141, 568–581. 10.1093/brain/awx353 29315361

[B31] Jack C. R. Jr. Lowe V. J. Weigand S. D. Wiste H. J. Senjem M. L. Knopman D. S.. (2009). Serial PIB and MRI in normal, mild cognitive impairment and Alzheimer’s disease: implications for sequence of pathological events in Alzheimer’s disease. Brain 132, 1355–1365. 10.1093/brain/awp062 19339253 PMC2677798

[B32] Jang Y. K. Lyoo C. H. Park S. Oh S. J. Cho H. Oh M.. (2018). Head to head comparison of [^18^F] AV-1451 and [^18^F] THK5351 for tau imaging in Alzheimer’s disease and frontotemporal dementia. Eur. J. Nucl. Med. Mol. Imaging 45, 432–442. 10.1007/s00259-017-3876-0 29143870

[B33] Jenkinson M. Bannister P. Brady M. Smith S. (2002). Improved optimization for the robust and accurate linear registration and motion correction of brain images. Neuroimage 17, 825–841. 10.1016/s1053-8119(02)91132-8 12377157

[B34] Jenkinson M. Beckmann C. F. Behrens T. E. Woolrich M. W. Smith S. M. (2012). FSL. Neuroimage 62, 782–790. 10.1016/j.neuroimage.2011.09.015 21979382

[B35] Kang J. M. Lee S. Y. Seo S. Jeong H. J. Woo S. H. Lee H.. (2017). Tau positron emission tomography using [^18^F]THK5351 and cerebral glucose hypometabolism in Alzheimer’s disease. Neurobiol. Aging 59, 210–219. 10.1016/j.neurobiolaging.2017.08.008 28890300

[B36] Leech R. Sharp D. J. (2014). The role of the posterior cingulate cortex in cognition and disease. Brain 137, 12–32. 10.1093/brain/awt162 23869106 PMC3891440

[B37] Lehmann M. Madison C. Ghosh P. M. Miller Z. A. Greicius M. D. Kramer J. H.. (2015). Loss of functional connectivity is greater outside the default mode network in nonfamilial early-onset alzheimer’s disease variants. Neurobiol. Aging 36, 2678–2686. 10.1016/j.neurobiolaging.2015.06.029 26242705 PMC4698410

[B38] Lemoine L. Gillberg P. G. Svedberg M. Stepanov V. Jia Z. Huang J.. (2017). Comparative binding properties of the tau PET tracers THK5117, THK5351, PBB3 and T807 in postmortem Alzheimer brains. Alzheimers Res. Ther. 9:96. 10.1186/s13195-017-0325-z 29229003 PMC5725799

[B39] Levitt P. Pintar J. E. Breakfield X. O. (1982). Immunocytochemical demonstration of monoamine oxidase B in brain astrocytes and serotonergic neurons. Proc. Natl. Acad. Sci. U S A 79, 6385–6389. 10.1073/pnas.79.20.6385 6755469 PMC347126

[B40] Leyns C. E. G. Holtzman D. M. (2017). Glial contributions to neurodegeneration in tauopathies. Mol. Neurodegener. 12:50. 10.1186/s13024-017-0192-x 28662669 PMC5492997

[B41] Lim H. K. Nebes R. Snitz B. Cohen A. Mathis C. Price J.. (2014). Regional amyloid burden and intrinsic connectivity networks in cognitively normal elderly subjects. Brain 137, 3327–3338. 10.1093/brain/awu271 25266592 PMC4240287

[B42] Lustig C. Snyder A. Z. Bhakta M. O’Brien K. C. McAvoy M. Raichle M. E.. (2003). Functional deactivations: change with age and dementia of the Alzheimer type. Proc. Natl. Acad. Sci. U S A 100, 14504–14509. 10.1073/pnas.2235925100 14608034 PMC283621

[B43] McKhann G. M. Knopman D. S. Chertkow H. Hyman B. T. Jack C. R. Jr. Kawas C. H.. (2011). The diagnosis of dementia due to Alzheimer’s disease: recommendations from the National Institute on Aging-Alzheimer’s Association workgroups on diagnostic guidelines for Alzheimer’s disease. Alzheimers Dement. 7, 263–269. 10.1016/j.jalz.2011.03.005 21514250 PMC3312024

[B44] Mioshi E. Dawson K. Mitchell J. Arnold R. Hodges J. R. (2006). The Addenbrooke’s cognitive examination revised (ACE-R): a brief cognitive test battery for dementia screening. Int. J. Geriatr. Psychiatry 21, 1078–1085. 10.1002/gps.1610 16977673

[B45] Moeller J. R. Strother S. C. (1991). A regional covariance approach to the analysis of functional patterns in positron emission tomographic data. J. Cereb. Blood Flow Metab. 11, A121–A135. 10.1038/jcbfm.1991.47 1997480

[B46] Montine T. J. Phelps C. H. Beach T. G. Bigio E. H. Cairns N. J. Dickson D. W.. (2012). National Institute on Aging-Alzheimer’s Association guidelines for the neuropathologic assessment of Alzheimer’s disease: a practical approach. Acta Neuropathol. 123, 1–11. 10.1007/s00401-011-0910-3 22101365 PMC3268003

[B47] Mormino E. C. Smiljic A. Hayenga A. O. Onami S. H. Greicius M. D. Rabinovici G. D.. (2011). Relationships between β-amyloid and functional connectivity in different components of the default mode network in aging. Cereb. Cortex 21, 2399–2407. 10.1093/cercor/bhr025 21383234 PMC3169663

[B48] Myers N. Pasquini L. Göttler J. Grimmer T. Koch K. Ortner M.. (2014). Within-patient correspondence of amyloid-β and intrinsic network connectivity in Alzheimer’s disease. Brain 137, 2052–2064. 10.1093/brain/awu103 24771519 PMC4065018

[B49] Ng K. P. Pascoal T. A. Mathotaarachchi S. Therriault J. Kang M. S. Shin M.. (2017). Monoamine oxidase B inhibitor, selegiline, reduces ^18^F-THK5351 uptake in the human brain. Alzheimers Res. Ther. 9:25. 10.1186/s13195-017-0253-y 28359327 PMC5374697

[B50] Nichols T. E. Holmes A. P. (2002). Nonparametric permutation tests for functional neuroimaging: a primer with examples. Hum. Brain Mapp. 15, 1–25. 10.1002/hbm.1058 11747097 PMC6871862

[B51] Niethammer M. Tang C. C. Feigin A. Allen P. J. Heinen L. Hellwig S.. (2014). A disease-specific metabolic brain network associated with corticobasal degeneration. Brain 137, 3036–3046. 10.1093/brain/awu256 25208922 PMC4208467

[B52] Okamura N. Harada R. Ishiki A. Kikuchi A. Nakamura T. Kudo Y. (2018). The development and validation of tau PET tracers: current status and future directions. Clin. Transl. Imaging 6, 305–316. 10.1007/s40336-018-0290-y 30148121 PMC6096533

[B53] Petersen R. C. (2004). Mild cognitive impairment as a diagnostic entity. J. Intern. Med. 256, 183–194. 10.1111/j.1365-2796.2004.01388.x 15324362

[B54] Ranasinghe K. G. Hinkley L. B. Beagle A. J. Mizuiri D. Dowling A. F. Honma S. M.. (2014). Regional functional connectivity predicts distinct cognitive impairments in Alzheimer’s disease spectrum. Neuroimage Clin. 5, 385–395. 10.1016/j.nicl.2014.07.006 25180158 PMC4145532

[B56] Rodríguez-Arellano J. J. Parpura V. Zorec R. Verkhratsky A. (2016). Astrocytes in physiological aging and Alzheimer’s disease. Neuroscience 323, 170–182. 10.1016/j.neuroscience.2015.01.007 25595973

[B58] Rowe C. C. Ng S. Ackermann U. Gong S. J. Pike K. Savage G.. (2007). Imaging β-amyloid burden in aging and dementia. Neurology 68, 1718–1725. 10.1212/01.wnl.0000261919.22630.ea 17502554

[B59] Sacks C. A. Avorn J. Kesselheim A. S. (2017). The failure of solanezumab-how the FDA saved taxpayers billions. N. Engl. J. Med. 376, 1706–1708. 10.1056/NEJMp1701047 28467878

[B60] Saxena S. Caroni P. (2011). Selective neuronal vulnerability in neurodegenerative diseases: from stressor thresholds to degeneration. Neuron 71, 35–48. 10.1016/j.neuron.2011.06.031 21745636

[B61] Serra L. Cercignani M. Mastropasqua C. Torso M. Spano B. Makovac E.. (2016). Longitudinal changes in functional brain connectivity predicts conversion to Alzheimer’s disease. J. Alzheimers Dis. 51, 377–389. 10.3233/JAD-150961 26890769

[B62] Sheline Y. I. Raichle M. E. Snyder A. Z. Morris J. C. Head D. Wang S.. (2010). Amyloid plaques disrupt resting state default mode network connectivity in cognitively normal elderly. Biol. Psychiatry 67, 584–587. 10.1016/j.biopsych.2009.08.024 19833321 PMC2829379

[B63] Shirer W. R. Ryali S. Rykhlevskaia E. Menon V. Greicius M. D. (2012). Decoding subject-driven cognitive states with whole-brain connectivity patterns. Cereb. Cortex 22, 158–165. 10.1093/cercor/bhr099 21616982 PMC3236795

[B66] Smith S. M. (2002). Fast robust automated brain extraction. Hum. Brain Mapp. 17, 143–155. 10.1002/hbm.10062 12391568 PMC6871816

[B65] Smith S. M. Nichols T. E. (2009). Threshold-free cluster enhancement: addressing problems of smoothing, threshold dependence and localization in cluster inference. Neuroimage 44, 83–98. 10.1016/j.neuroimage.2008.03.061 18501637

[B67] Snowdon D. A. (1997). Aging and Alzheimer’s disease: lessons from the Nun study. Gerontologist 37, 150–156. 10.1093/geront/37.2.150 9127971

[B68] Song Z. Insel P. S. Buckley S. Yohannes S. Mezher A. Simonson A.. (2015). Brain amyloid-β burden is associated with disruption of intrinsic functional connectivity within the medial temporal lobe in cognitively normal elderly. J. Neurosci. 35, 3240–3247. 10.1523/JNEUROSCI.2092-14.2015 25698758 PMC4331637

[B69] Sperling R. A. Laviolette P. S. O’Keefe K. O’Brien J. Rentz D. M. Pihlajamaki M.. (2009). Amyloid deposition is associated with impaired default network function in older persons without dementia. Neuron 63, 178–188. 10.1016/j.neuron.2009.07.003 19640477 PMC2738994

[B70] Spetsieris P. Ma Y. Peng S. Ko J. H. Dhawan V. Tang C. C.. (2013). Identification of disease-related spatial covariance patterns using neuroimaging data. J. Vis. Exp. 76:e50319. 10.3791/50319 23851955 PMC3728991

[B71] Stam C. J. (2014). Modern network science of neurological disorders. Nat. Rev. Neurosci. 15, 683–695. 10.1038/nrn3801 25186238

[B72] Utevsky A. V. Smith D. V. Huettel S. A. (2014). Precuneus is a functional core of the default-mode network. J. Neurosci. 34, 932–940. 10.1523/jneurosci.4227-13.2014 24431451 PMC3891968

[B73] Verdurand M. Bort G. Tadino V. Bonnefoi F. Le Bars D. Zimmer L. (2008). Automated radiosynthesis of the Pittsburg compound-B using a commercial synthesizer. Nucl. Med. Commun. 29, 920–926. 10.1097/MNM.0b013e328304e0e1 18769311

[B74] Villemagne V. L. Burnham S. Bourgeat P. Brown B. Ellis K. A. Salvado O.. (2013). Amyloid β deposition, neurodegeneration and cognitive decline in sporadic Alzheimer’s disease: a prospective cohort study. Lancet Neurol. 12, 357–367. 10.1016/S1474-4422(13)70044-9 23477989

[B75] Villemagne V. L. Pike K. E. Chételat G. Ellis K. A. Mulligan R. S. Bourgeat P.. (2011). Longitudinal assessment of Aβ and cognition in aging and Alzheimer disease. Ann. Neurol. 69, 181–192. 10.1002/ana.22248 21280088 PMC3045039

[B77] Wang Y. Yan T. Lu H. Yin W. Lin B. Fan W.. (2017). Lessons from anti-amyloid-β immunotherapies in Alzheimer disease: aiming at a moving target. Neurodegener. Dis. 17, 242–250. 10.1159/000478741 28787714

[B76] Wang P. Zhou B. Yao H. Zhan Y. Zhang Z. Cui Y.. (2015). Aberrant intra- and inter-network connectivity architectures in Alzheimer’s disease and mild cognitive impairment. Sci. Rep. 5:14824. 10.1038/srep14824 26439278 PMC4594099

[B79] Wu J. W. Hussaini S. A. Bastille I. M. Rodriguez G. A. Mrejeru A. Rilett K.. (2016). Neuronal activity enhances tau propagation and tau pathology *in vivo*. Nat. Neurosci. 19, 1085–1092. 10.1038/nn.4328 27322420 PMC4961585

[B80] Zhang S. Li C. S. (2012). Functional connectivity mapping of the human precuneus by resting state fMRI. Neuroimage 59, 3548–3562. 10.1016/j.neuroimage.2011.11.023 22116037 PMC3288461

