# Supplementary figures and images for: Involvement of the Precuneus/Posterior Cingulate Cortex Is Significant for the Development of Alzheimer’s Disease: A PET (THK5351, PiB) and Resting fMRI Study
Source: Front Aging Neurosci. 2018 Oct 5;10:304. doi: 10.3389/fnagi.2018.00304 (PMC6182068; doi:10.3389/fnagi.2018.00304)

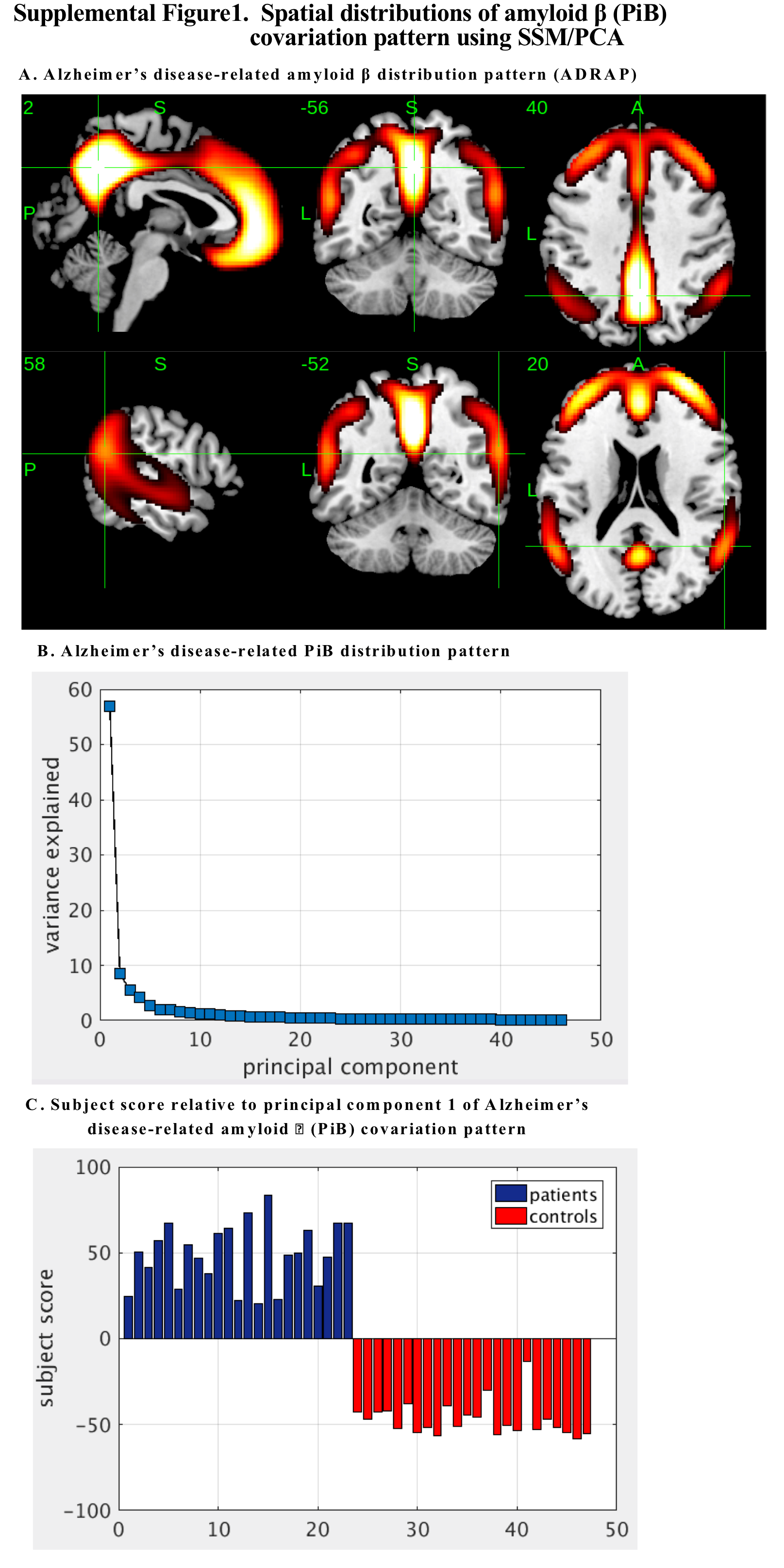

Supplement: FIGURE S1 — Spatial distributions of the amyloid β (PiB) covariation pattern using SSM/PCA. Panel (A) shows the Alzheimer’s disease-related Aβ distribution pattern (ADRAP). SSM/PCA identified an Alzheimer’s disease-related Aβ covariation pattern (ADRAP). The hot areas represent principal component 1 of ADRAP. Panel (B) is the Alzheimer’s disease-related PiB distribution pattern. This figure indicates the ratio of each principal component with the total subject voxel variance of data. Panel (C) is Subject score relative to principal component 1 of Alzheimer’s disease-related amyloid β (PiB) covariation pattern. [file Image_1.TIFF]
